# The flavonoid, fisetin, inhibits UV radiation-induced oxidative stress and the activation of NF-кB and MAPK signaling in human lens epithelial cells

**Published:** 2008-10-20

**Authors:** Ke Yao, Li Zhang, YiDong Zhang, PanPan Ye, Ning Zhu

**Affiliations:** Eye Center, Affiliated Second Hospital, College of Medicine, Zhejiang University, Hangzhou, China

## Abstract

**Purpose:**

Ultraviolet (UV) radiation-induced oxidative stress plays a significant role in the progression of cataracts. This study investigated the photoprotective effect of fisetin on UV radiation-induced oxidative stress in human lens epithelial cells and the possible molecular mechanism involved.

**Methods:**

SRA01/04 cells exposed to different doses of ultraviolet B (UVB) were cultured with various concentrations of fisetin and subsequently monitored for cell viability by the 4,5-dimethylthiazol-2-yl-2,5-diphenyltetrazolium bromide (MTT) assay. The effect of fisetin on the generation of reactive oxygen species (ROS) of SRA01/04 cells was determined by flow cytometry. Translocation of nuclear factor kappa-B (NF-кB) was examined by immunocytochemistry. Expression of NF-кB/P65, inhibiter kappa B (IкB), and mitogen activated protein kinase (MAPK) proteins were measured by western blot.

**Results:**

Treatment of SRA01/04 cells with fisetin inhibited UVB-induced cell death and the generation of ROS. Fisetin inhibited UVB-induced activation and translocation of NF-кB/p65, which was mediated through an inhibition of the degradation and activation of IкB. Fisetin also inhibited UVB-induced phosphorylation of the p38 and c-Jun N-terminal kinase (JNK) proteins of the MAPK family at various time points studied.

**Conclusions:**

The flavonoid, fisetin, could be useful in attenuation of UV radiation-induced oxidative stress and the activation of NF-кB and MAPK signaling in human lens epithelial cells, which suggests that fisetin has a potential protective effect against cataractogenesis.

## Introduction

Cataracts are the main cause of human blindness worldwide, responsible for 48% of the total cases of blindness [1,2]. Understanding the pathophysiology of cataract formation is important not only to advance the state of medical knowledge but also for public health purposes. Ultraviolet (UV) irradiation is reportedly the most closely associated factor in epidemiologic and experimental studies [3-6]. Several studies have shown that exposure of lens epithelial cells to physiologic doses of UV increases reactive oxygen species (ROS) production and oxidative stress, which results from ROS as the major mechanism of cellular damage and cataractogenesis [6-9].

UV irradiation leads to the formation of ROS, which results in the subsequent activation of complex signaling pathways including nuclear factor kappa-B (NF-кB) and mitogen activated protein kinase (MAPK) pathways [10,11]. NF-кB is a ubiquitous transcription factor. It is a multiprotein complex that can activate a great variety of genes involved in early defensive reactions of higher organisms. It has been demonstrated that NF-кB plays an important role in cellular death, which takes place after UV irradiation [12,13].

Epidemiologic data have indicated that certain dietary additives can help provide an effective defense against oxidative stress and thus have potential in the treatment of a variety of diseases. Flavonoids are a class of natural biological products that have evolved to protect plants from the oxidative damage induced by chronic exposure to ultraviolet light. Many flavonoids act directly as antioxidants, neutralizing toxic ROS by donating hydrogen ions [14]. Fisetin (3, 3′, 4’, 7-tetrahydroxyflavone) is a flavonoid dietary ingredient widely distributed in fruits and vegetables such as strawberries, apples, persimmons, grapes, onions, and cucumbers at concentrations of 2-160 μg/g [15]. It exhibits a wide variety of activities including neurotrophic, antioxidant, anti-inflammatory, and antiangiogenic effects [16-18]. Recently, fisetin along with luteolin, quercetin, eriodictyol, baicalein, galangin, and epigallocatechin gallate (EGCG) was found to protect human retinal pigment epithelial (RPE) cells from oxidative stress-induced death with a high degree of potency and low toxicity [19]. There is no study about the effect of fisetin on UV radiation-induced oxidative stress and the precise mechanism of signal transduction in human lens epithelial (HLE) cells.

Based on these recent studies, we hypothesized that fisetin would protect HLE cells from oxidative stress by influencing several signaling pathways and hence would be beneficial in the treatment of cataract. To test this hypothesis, we used UV-exposed HLE cells as a model in vitro. This study is designed to investigate the protective effect of fisetin against UV radiation-induced oxidative stress in HLE cells along with the mechanism involved.

## Methods

### Materials

The human lens epithelial cell line, SRA01/04 [20], was obtained from the Riken Cell Bank (Tsukuba, Japan). Fetal bovine serum (FBS) and Dulbecco's modified Eagle's medium (DMEM) were obtained from Gibco (Grand Island, NY). Fisetin, dimethylsulfoxide (DMSO), 4,5-dimethylthiazol-2-yl-2,5-diphenyltetrazolium bromide (MTT), dichlorofluorescein diacetate (DCF-DA), and a protease inhibitor cocktail were purchased from Sigma Chemical Co. (St. Louis, MO). Fisetin was dissolved in DMSO to 100 mM. The BCA protein assay kit was from Pierce (Lockford, IL). The nuclear extract kit was obtained from Active Motif (Carlsbad, CA). The enhanced chemiluminescence (ECL) detection kit was acquired from Amersham Pharmacia (Arlington Heights, IL). Anti-p65, anti-inhibitor kappa B (IкB), anti-actin, Cy-3 conjugated goat anti-rabbit IgG, anti-mouse, and anti-rabbit IgG horseradish peroxidase (HRP) antibodies were purchased from Santa Cruz Biotechnology, Inc. (Santa Cruz, CA). Anti-p-extracellular signal-regulated kinase (ERK1/2), anti-ERK1/2, anti-p- c-Jun N-terminal kinase (JNK), anti-JNK, anti-p-p38, and anti-p38 antibodies were obtained from Cell Signaling (Beverly, MA).

### Cell culture and ultraviolet irradiation

The SRA01/04 cells were seeded at a density of 2×10^6^/dish in 60-mm dishes (Falcon; Becton-Dickinson, Oxnard, CA) in DMEM with 10% heat-inactivated (56 °C for 0.5 h) fetal FBS at 37 °C in a humidified atmosphere of 5% CO_2_. Cells were subcultured twice a week, and only those in the exponential growth phase were used in experiments. Fisetin was added at the indicated doses less than 0.1% DMSO at the indicated time points. Ultraviolet B (UVB) irradiation was performed using a UV lamp (CL-1000M, UVP, Upland, CA) monitored with a UVX radiometer (UVP). The majority of resulting wavelengths were in UVB range (290–320 nm), and the peak emission was recorded at 302 nm. Prior to irradiation, cells were washed twice in warm PBS (pH 7.4) and then replaced with PBS. Various UVB energy sources (0, 30, 60, 90 mJ/cm^2^) were irradiated on SRA01/04 cells. Following irradiation, the cells were washed twice in warm PBS, and the growth medium was replaced and further incubated for 0 h, 3 h, 6 h, 12 h, 24 h, and 48 h. A fixed total exposure of 30 mJ/cm^2^ was used for the subsequent exam with or without fisetin pretreatment.

### Measurement of cell viability

The MTT assay, which measures cell proliferation and cytotoxicity, was used to verify the viability of SRA01/04 cells. Cells were incubated with different concentrations of fisetin (0, 5, 10, 25, 50, 100 μg/ml) for 1 h and further incubated for 24 h after exposure. Cells were incubated with 20 μl of MTT solution (0.5 mg/ml) for 4 h at 37 °C, and the solution was then replaced with 200 μl DMSO after the incubation. The absorbance was measured at 490 nm by a microplate reader (ELx800; BioTek, Winooski, VT).

### Flow cytometry analysis

Production of reactive oxygen species (ROS) was monitored by flow cytometry. Cells were seeded onto chamber slides and grown until confluent. After exposure, DCF-DA (10 μM) was added into the medium for 15 min at 37 °C. Data was then acquired on a Cytomics FC500 flow cytometer (Beckman Coulter, Fullerton, CA) with excitation at 480 nm and emission at 530 nm. Results were analyzed using CXP software (Beckman Coulter).

### Immunocytochemistry

Translocation of NF-кB p65 was monitored immunocytochemically. Cells were seeded onto chamber slides and grown until confluent. After exposure, slides were washed twice with ice-cold PBS followed by fixation with 100% ethanol for 5 min at 4 °C. Slides were then incubated overnight at 4 °C with anti-p65 antibody (1:500) and incubated in Cy-3 conjugated goat anti-rabbit IgG (1:500) for 30 min at room temperature. Stained slides were examined by fluorescence microscopy (Olympus, Tokyo, Japan). After exposure, DCFH-DA (10 μM) was added into the medium for 15 min at 37 °C to monitor the generation of ROS. Stained slides were examined by fluorescence microscopy.

### Western blot analysis

After treatment, cultured cells were washed with cold PBS and then lysed in a buffer containing 50 mM Tris-HCl, pH 7.5, 150 mM NaCl, 1 mM Na_2_EDTA, 1 mM EDTA, 1% Triton-X100, 2.5 mM sodium pyrophosphate, 1 mM β-glycerophosphate, 1 mM Na_3_VO_4_, 1 mM NaF, 1 μg/ml leupeptin, and 1 mM phenylmethanesulfonyl fluoride (PMSF). Nuclear extracts were collected according to the instruction of the nuclear extract kit. Protein extracts were quantified using the BCA protein assay kit. Equal amounts of protein (50 µg/lane) were resolved by 10% sodium dodecyl sulfate-polyacrylamide gel electrophoresis (SDS–PAGE) and transferred onto a polyvinylidene difluoride (PVDF) membrane (Millipore, Milford, MA). Blots were blocked for 1 h in blocking buffer (PBS with 7.5% non-fat dry milk, 2% BSA, 0.1% Tween) and incubated with primary antibodies (1:1000) overnight at 4 °C. Membranes were washed subsequently in washing buffer (tris-buffered saline tween-20 [TBST] with 0.1% Tween-20) and incubated with HRP-conjugated secondary antibodies for 1 h at room temperature. Membranes were then detected by ECL western detection reagents.

### Statistical analysis

Values were expressed as the mean±SD of at least three independent experiments and analyzed with one-way ANOVA. p values less than 0.05 values were considered statistically significant.

## Results

### Effect of fisetin on the viability of UVB-exposed human lens epithelial cells

**Figure 1:**
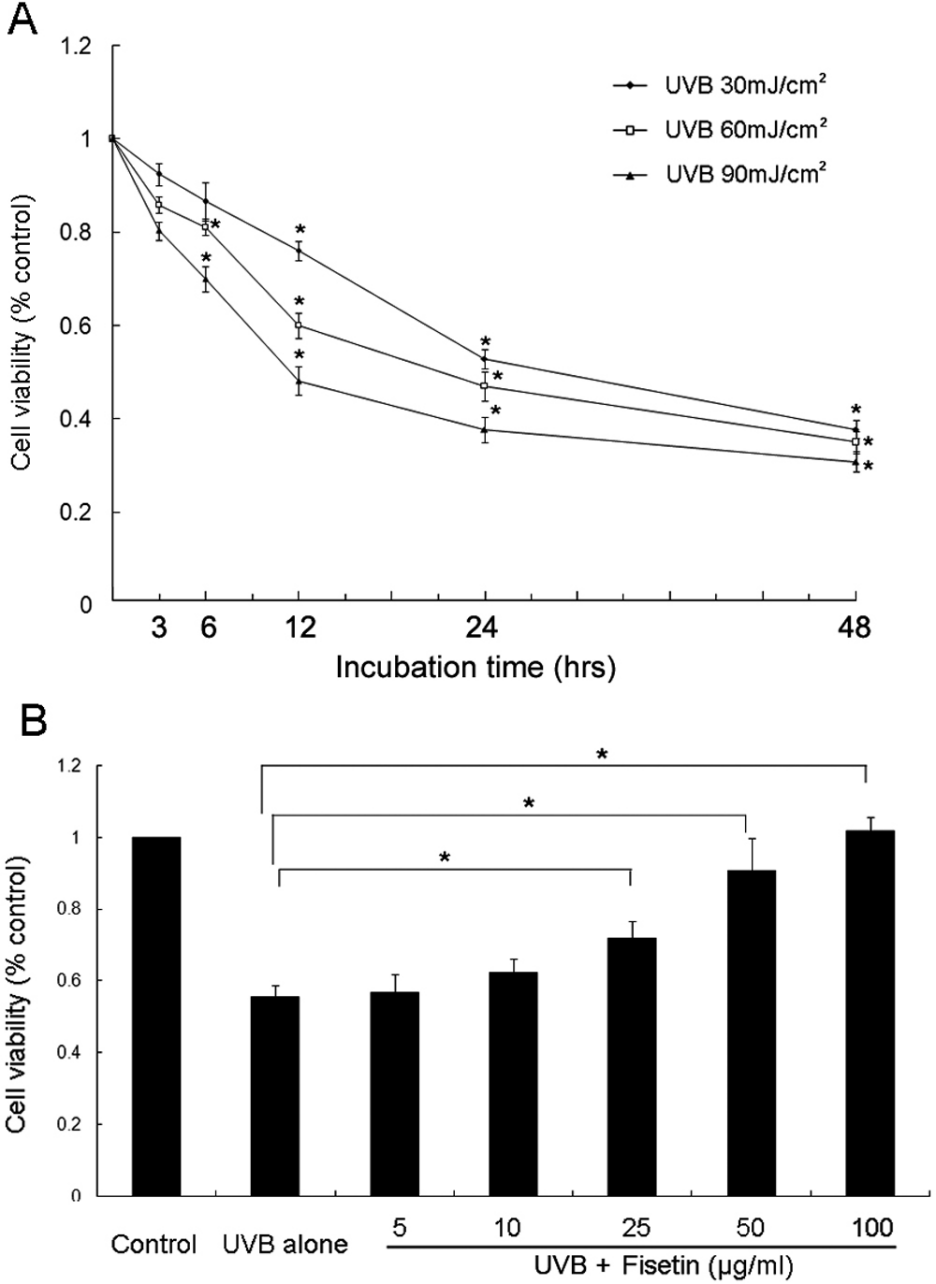
Effect of fisetin on the viability of UVB-exposed human lens epithelial cells. **A**: Cell viability of SRA01/04 to different irradiation intensities of UVB (30, 60, and 90 mJ/cm^2^) is shown at different incubation times of 0, 3, 6, 12, 24, and 48 h. The viability was measured by MTT assay. **B**: The cell viability of cells pretreated with fisetin at different concentrations (0, 5, 10, 25, 50, and 100 μg/ml) for 1 h before exposure to 30 mJ/cm^2^ UVB was estimated using MTT assay after being cultured for 24 h. Data are represented as the mean±SD from three independent experiments. An asterisk indicates p<0.05.

As shown in Figure 1, UVB irradiation produced a progressive cytotoxic effect on cultured SRA01/04 cells in a dose-dependent manner (Figure 1A). UVB irradiation at 30 mJ/cm^2^ significantly reduced cell viability in a time-dependent manner. The irradiation energy of 30 mJ/cm^2^ was thus adopted for further studies. When fisetin, in concentrations between 5 and 100 μg/ml, was added to UVB-exposed cells for 24 h, the viability was substantially enhanced in a dose-dependent manner (Figure 1B). Pretreatment of 25 μg/ml fisetin significantly inhibited UVB-induced cell damage (p<0.05; Figure 1B).

### Fisetin inhibits the UVB-induced generation of reactive oxygen species in human lens epithelial cells

**Figure 2:**
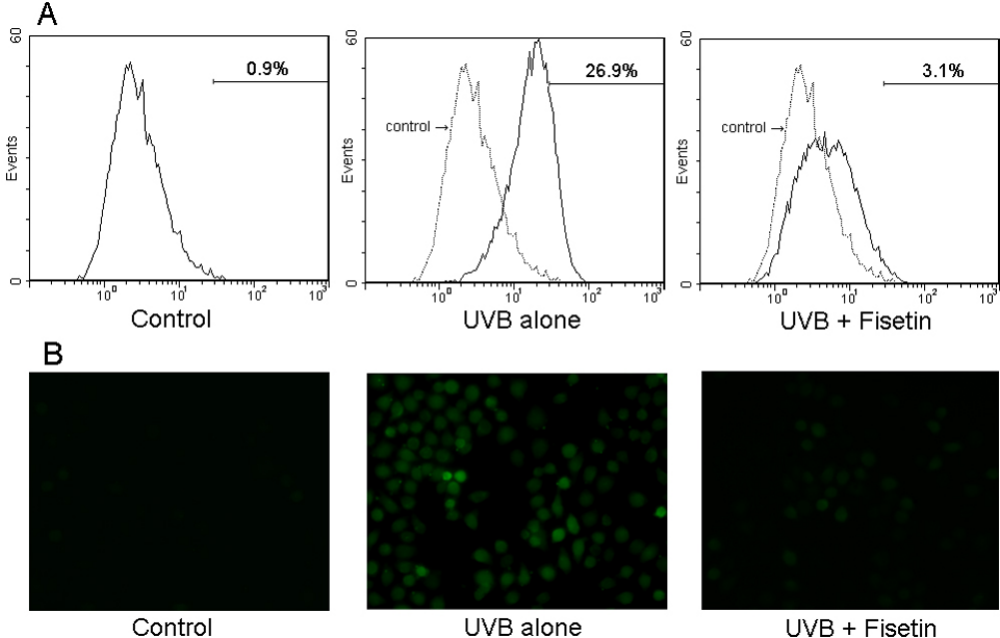
Effect of fisetin on UVB-induced generation of reactive oxygen species in human lens epithelial cells. **A**: SRA01/04 cells were pretreated with 25 μg/ml fisetin for 1 h followed by exposure to 30 mJ/cm^2^ UVB before incubation for 2 h and being loaded with DCFH-DA. Oxidant generation was measured by DCF flow cytometry as described in Methods. **B**: Representative fluorescent images of controls and UVB-irradiated cells were taken under fluorescence microscopy. Magnification, 100X.

DCF-DA was used to carry out the generation of ROS. As shown in Figure 2A, treatment with 30 mJ/cm^2^ UVB significantly enhanced the generation of ROS from 0.9% to 26.9%. Pretreatment with 25 μg/ml fisetin distinctly reduced the generation of ROS. The generation of ROS was also examined by DCF fluorescence. There was an expected lack of staining in the UVB-free control group (Figure 2B). SRA01/04 cells exposed to UVB alone had markedly heavy staining, indicating a marked increase in ROS generation at the single cell level. UVB-exposed cells revealed a significant decrease of DCF staining in the presence of fisetin (Figure 2B).

### Fisetin inhibits UVB-induced activation and translocation of NF-кB in human lens epithelial cells

**Figure 3:**
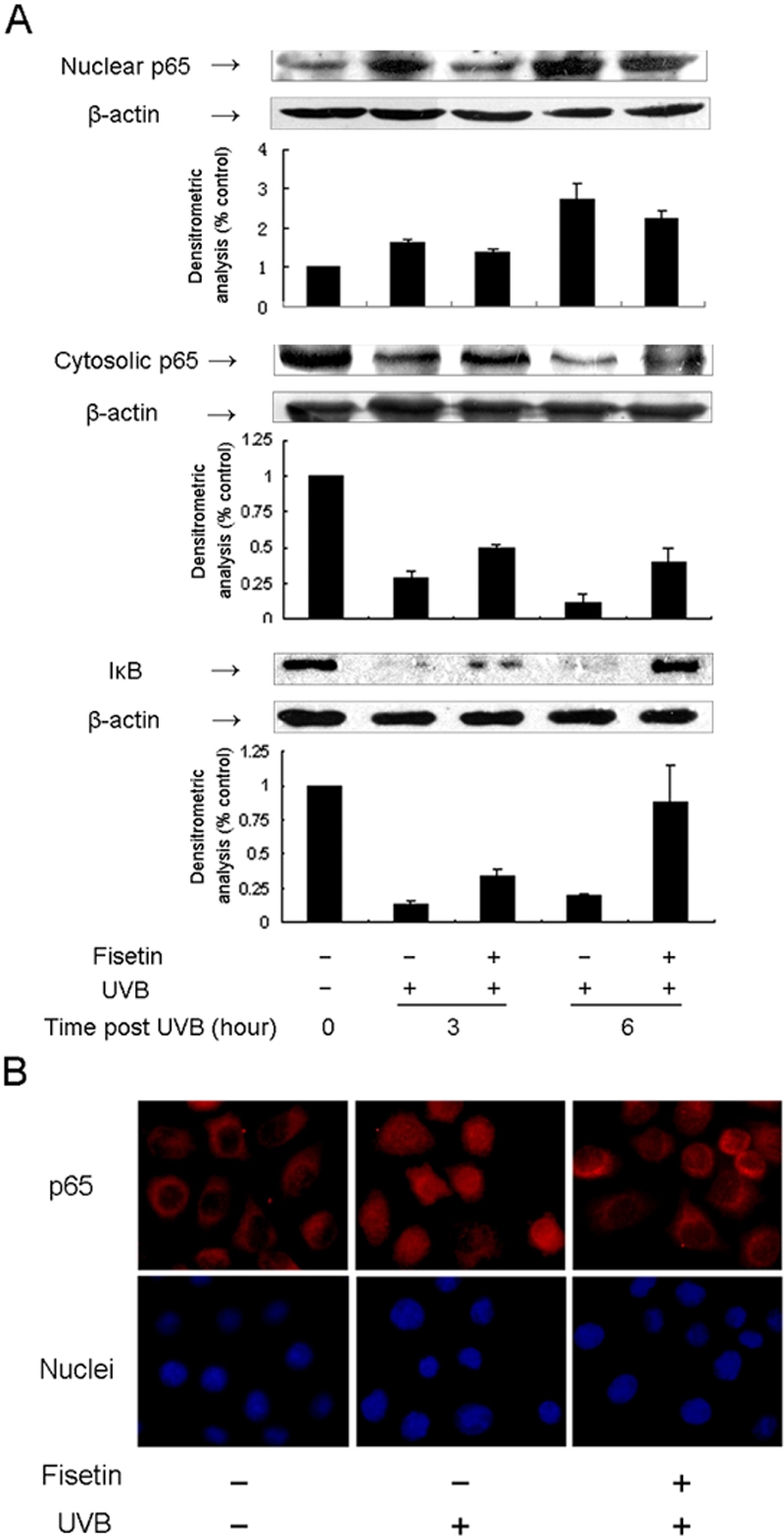
Effect of fisetin on UVB-induced activation of NF-кB and degradation of IкB in HLE cells. **A**: SRA01/04 cells were exposed to UVB (30 mJ/cm^2^) with or without pretreatment with fisetin (25 μg/ml) for 1 h. Cells were harvested at 3 h and 6 h time points after UVB exposure, and cell lysates were prepared to determine the activation of NF-кB or degradation of IкB using western blot analysis. The graph represents the quantification results normalized to β-actin levels. Data represent the mean±SD of three individual experiments. The asterisk indicates p<0.05. **B**: Immunocytochemical analysis of NF-кB p65 localization is visualized. Cultured cells were incubated with anti-p65 antibody overnight at 4 °C as described in Methods. p65 is stained red, and the nuclei are stained blue. Representative fluorescent images were taken under fluorescence microscopy. Magnification, 100X.

Activation of NF-кB was based on the detection of its translocation into cell nuclei from its initial location in the cytoplasm where it exists in an inactive form. Cells exposed to UVB exhibited an enhancement of nuclear NF-кB/p65 and a reduction of cytosolic NF-кB /p65 3 h after irradiation, and this became more evident 6 h after irradiation (Figure 3A). Western blot indicated that treatment with fisetin before UVB irradiation markedly abrogated UVB-induced activation of NF-кB/p65 in a time-dependent manner. Similarly, immunocytochemical studies showed NF-кB/p65 primarily resided in the cytoplasm and translocated into nuclei 3 h after exposure. In contrast, NF-кB/p65 mostly remained in the cytoplasm with scant translocation found in the cells treated with fisetin (Figure 3B). We determined whether UVB-induced degradation of IкB is inhibited by fisetin treatment, which inhibits the activation and translocation of NF-кB. Western blot indicated that exposure to UVB radiation resulted in a degradation of the IкB protein at each time point studied compared to non-UVB-exposed cells (Figure 3B). The UVB-induced degradation of IкB was inhibited after 3 h and almost completely inhibited 6 h after exposure in the cells pretreated with fisetin (Figure 3A).

### Effect of fisetin on UVB-induced phosphorylation of the mitogen activated protein kinase pathway

**Figure 4:**
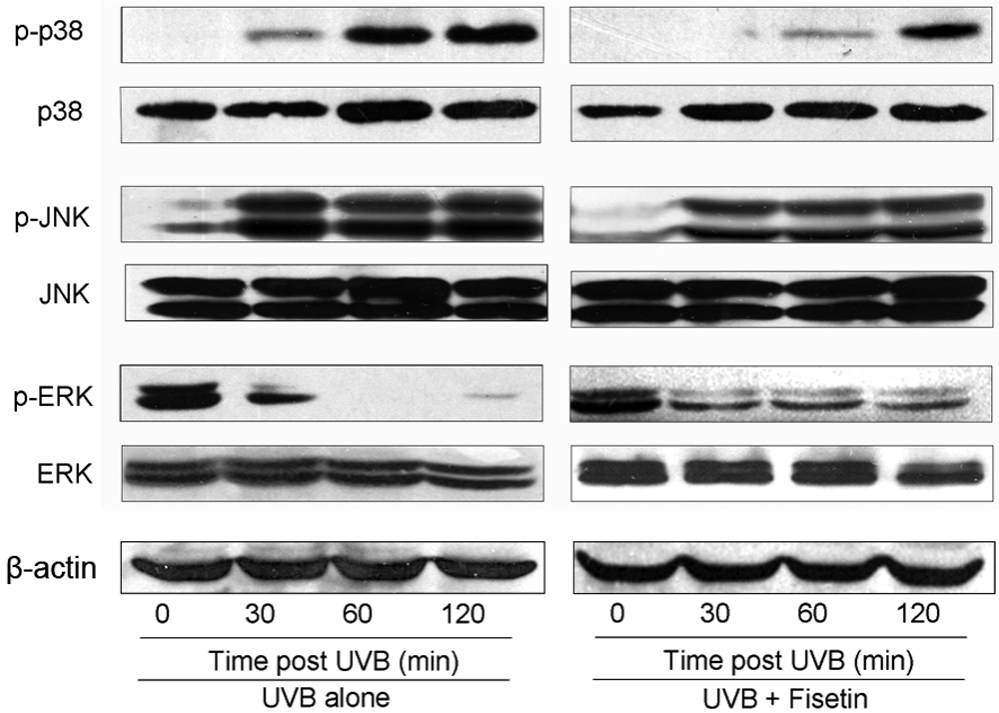
Effect of fisetin on UVB-induced phosphorylation of the MAPK pathway. SRA01/04 cells were exposed to UVB (30 mJ/ cm^2^) with or without pretreatment with fisetin (25 μg/ml) for 1 h. Cells were harvested at different time points (30, 60, and 120 min) after UVB exposure, and cell lysates were prepared. The phosphorylated and total protein levels of ERK1/2, JNK, and p38 were detected with specific antibodies using western blot analysis as described in Methods. A representative blot from three independent experiments with identical observations is shown.

Data on the kinetics of MAPK activation in UVB-irradiated cells showed that phosphorylation of p38 started 30 min after irradiation and that maximum phosphorylation occurred 2 h after irradiation (Figure 4). Western blot and subsequent measurement of the intensity of the bands relative to the total amount of p38 phosphorylation indicated that treatment with fisetin markedly inhibited UVB-induced phosphorylation of p38 at each time point studied. A marked induction in JNK phosphorylation started after 30 min and remained at a high level until 2 h had passed (Figure 4). Treatment with fisetin inhibited UVB-induced phosphorylation of JNK at each time point studied. In contrast, exposure of SRA01/04 cells to UVB reduced phosphorylation of ERK1/2 (p42/p44) after 30 min, and the reduced levels of ERK1/2 phosphorylation were observed until 2 h after irradiation (Figure 4). Treatment with fisetin elevated the phosphorylation of ERK1/2 at each time point studied. Importantly, treatment of SRA01/04 cells with fisetin alone did not induce the phosphorylation of the ERK1/2, JNK, or p38 proteins of the MAPK family (data not shown). Further, the total amount of ERK1/2, JNK, and p38 remained unchanged at each time point studied.

## Discussion

UV-associated ocular damage has been of increasing interest to clinicians and researchers [4,21,]. It has been recognized that UVB, a 290–320 nm component of solar ultraviolet radiation, which induces oxidative stress both in vitro and in vivo, contributes to several adverse biological effects on the lens. There is considerable evidence that the cellular damage effect of UVB radiation is mediated through the UVB-induced, oxidative stress-mediated activation of signal transduction pathways that control gene expression [22,23]. Flavonoids can provide both short-term and long-term protection against oxidative stress through a variety of mechanisms. Many flavonoids act directly as antioxidants, neutralizing toxic ROS by donating hydrogen ions [14]. Yet of equal and potentially even greater importance, flavonoids can modulate cell signaling pathways [24]. As the cellular damage effect of UV radiation is mediated through the induction of oxidative stress, we determined the effect of fisetin on UVB-induced oxidative stress and its effect on the activation of the MAPK and NF-кB signaling pathways. The results suggest that fisetin is a potent inhibitor of UV-induced oxidative stress and the activation of NF-кB and MAPK signaling in human lens epithelial cells.

Fisetin, a flavonoid compound with high trolox equivalent antioxidative capacity (TEAC) values, is hydrophobic and readily passes through cell membranes and accumulates intracellularly, resulting in a good antioxidant activity [25]. A previous study has shown that low concentrations of flavonoids are protective in cells whereas high concentrations cause DNA damage and apoptosis [26]. Fisetin-induced cytotoxity, DNA strand breaks, oligonucleosomal DNA fragmentation, and caspase-3 activation on their own occur at concentrations between 50 and 250 μmol/l. On the other hand, 50 μmol/l fisetin significantly protected against the large number of DNA strand breaks caused by 500 μmol/l H_2_O_2_ [26]. We did not observe this trend at the maximum concentration of 100 μg/ml in SRA01/04 cells. Therefore, it is worthwhile to determine the relative margin of exposure required for the respective cytoprotective and cytotoxic actions of fisetin in an HLE cell culture system. Additional pharmacokinetic investigations will be required to determine the suggested dietary supplement of fisetin, which would result in a proper plasma or aqueous humor concentration in human beings.

Oxidative stress could disrupt the balance between reactive oxygen radical production and the radical scavenging effect and lead to cell damage. The generation of ROS increased at the early stage of oxidative stress before cell morphological changes could be observed and cell death was evident in this study, indicating that ROS generation is early and a critical event in the death mechanism. The overproduction of ROS results in the subsequent activation of complex signaling pathways. To investigate the protective effect of fisetin against oxidative stress in HLE cells, we looked into the potential pathway involved. NF-кB is one of the most ubiquitous transcription factors. In unstimulated cells, NF-кB resides in the cytoplasm in an inactive complex with inhibitor kappa B (IкB). Pathogenic stimuli induce phosphorylation and the subsequent release of IкB, resulting in NF-кB translocation to the nucleus where it binds to DNA control elements and thus influences the transcription of certain specific genes [27,28]. The activation of NF-кB has an important regulatory role in inflammation, cell proliferation, and oncogenesis [29,30]. Therefore, the signaling pathways leading to the regulation of NF-кB activity have become a focal point of drug discovery efforts. NF-кB is commonly activated by agents that generate ROS such as UV radiation [31]. Based on the results obtained in most of the cell systems studied to date, the current model for NF-кB activation involves the degradation of IкB inhibitory proteins by the 26S proteasome, which allows for the translocation of NF-кB into the nucleus and the activation of NF-кB-inducible genes. Agents that scavenge ROS could inhibit the activation of NF-кB [32]. In this study, we observed that in vitro treatment with fisetin resulted in the prevention of a UVB-induced generation of ROS in SRA01/04 cells. Therefore, this observation provides a possible mechanism for the photoprotective effect of fisetin. In our study, NF-кB is activated by UVB irradiation and subsequently translocated into the nucleus. However, the activation and translocation were effectively inhibited by pretreatment with fisetin. UVB exposure also resulted in an increased degradation of IкB protein but the pretreatment of fisetin blocked this degradation.

A growing body of literature suggests that transient increases in ROS levels act as an important mediator of proliferation and results in the activation of various signaling molecules and pathways, including the MAPK pathway [33]. The MAPK pathway plays a crucial role in cellular responses such as proliferation, differentiation, and apoptosis. JNK and p38 are known as stress activated protein kinases (SAPK) and play key roles in cellular stress, apoptosis, and inflammation [34,35,]. Previous studies in our laboratory have shown that oxidative stress modulates the level of phosphorylated MAPKs including ERK1/2, JNK, and p38, which has been proved to play a role in cataractogenesis [36]. To investigate the photoprotective effect of fisetin, we determined its effect on UVB-induced phosphorylation of MAPK proteins using western blot analysis. Fisetin inhibited UVB irradiation-induced phosphorylation of p38 and JNK at different time points in our study. Fisetin also inhibited UVB irradiation-induced downregulation of p-ERK1/2. Thus, the photopreventive effect of fisetin may be associated with the inhibition of UVB-induced, oxidative stress-mediated activation of these MAPK pathways in this in vitro model. Previous studies have shown ERK and p38 proteins of the MAPK family to be involved in the activation of NF-кB [37-39]. Indeed, the exact mechanism of the inhibition of UVB-induced phosphorylation of MAPK proteins by fisetin is not clear based on the present data. It appears that the antioxidant property of fisetin contributed to the inhibition of the UVB-induced phosphorylation of MAPKs through both a modulation of ROS and prevention of downstream events such as NF-кB activation. Therefore, the inhibition of the MAPK and NF-кB signaling pathways could potentially be used by fisetin to activate certain antioxidant-responsive, element-dependent genes to protect against the UVB-induced oxidative stress in HLE cells.

In summary, the flavonoid, fisetin, protects HLE cells from UVB-induced oxidative stress by inhibiting the generation of ROS and modulating the activation of NF-кB and MAPK pathways. Fisetin possesses a potential pharmacological application in attenuating UVB radiation-induced oxidative stress, suggesting a protective effect against cataractogenesis.
